# Effect of personalized blood pressure management during mechanical thrombectomy under general anesthesia: a single-center before–after study

**DOI:** 10.1007/s00234-025-03878-6

**Published:** 2026-01-14

**Authors:** Vincent L’Allinec, Océane Palka, Madjid Bouizegarene, Lucas Dabouineau, Sophie Godard, Bertrand Lapergue, Sigismond Lasocki, Emmanuel Rineau, Maxime Léger

**Affiliations:** 1 Department of Radiology, University hospital of Angers, ANGERS, France; 2 Department of Anesthesiology and Intensive care, University hospital of Angers, ANGERS, France; 3 Department of Neurology and Stroke Unit, University hospital of Angers, ANGERS, France; 4 Neurology Department, Foch Hospital, Suresnes, France; 5 INSERM UMR 1246-SPHERE, University Nantes and Tours, Tours, Nantes, France

**Keywords:** Acute ischemic stroke, Mechanical thrombectomy, General anesthesia, Arterial blood pressure, Neurological outcome, Modified rankin scale

## Abstract

**Introduction:**

Blood pressure (BP) variability, particularly arterial hypotension during mechanical thrombectomy (MT) for acute ischemic stroke (AIS), has been associated with unfavorable outcomes. This variability in BP is notably present during induction in general anesthesia (GA). This study seeks to assess the effectiveness of a personalized BP management protocol during MT under general GA in mitigating hypotension and its impact on functional outcomes at 90 days.

**Methods:**

We conducted a monocentric before-after study involving two retrospective cohorts of patients who underwent MT for AIS under GA, before and after implementing the protocol. The protocol aimed to maintain the mean arterial pressure (MAP) within 10% of the baseline MAP prior to induction. The main outcome measured was the modified Rankin Scale (mRS) at 90 days.

**Results:**

Our analysis included 179 patients: 121 before and 58 after protocol implementation. The “after” group showed a reduced proportion of MAP drops, especially severe hypotension (32.8% vs. 48.8% below 30% from baseline, and 51.7% vs. 66.1% below 20%). However, these differences lacked statistical significance. At 90 days, a poor outcome (mRS > 2) was observed in 56.9% of patients in the ‘after’ group vs. 46.3% in the ‘before’ group, not statistically significant. This association remained statistically insignificant in both univariate and multivariate analyses.

**Conclusion:**

The personalized BP management during MT resulted in a decrease in BP drops without reaching significance. Furthermore, this study did not indicate any improvement in neurological outcomes at 90 days.

**Supplementary Information:**

The online version contains supplementary material available at 10.1007/s00234-025-03878-6.

## Highlights

Despite the lack of statistical significance, the use of a personalized blood pressure management protocol during mechanical thrombectomy under general anesthesia reduces the incidence of blood pressure drops.

In this single-center before-and-after study, the use of a personalized blood pressure protocol during mechanical thrombectomy under general anesthesia did not show an effect on patient outcomes at 3 months.

**Supplementary Information:**

The online version contains supplementary material available at 10.1007/s00234-025-03878-6.

## Introduction

Mechanical thrombectomy (MT), with or without intravenous thrombolysis (IVT), has become the established approach for treating acute ischemic stroke (AIS) caused by anterior large vessel occlusion (LVO) [1, 2]. Even with high recanalization rates, more than 50% of revascularized patients still face unfavorable outcomes at 90 days [3]. 

Among the potential factors contributing to these outcomes, prior research has highlighted the significant influence of blood pressure (BP) during MT. Multiple studies have identified an association between arterial hypotension and BP variability during MT and poorer functional outcomes at 90 days, both under general anesthesia (GA) and conscious sedation [4–9]. 

The ideal BP thresholds to uphold during MT remain undefined. Presently, the 2019 AHA/ASA stroke guidelines solely advise keeping systolic blood pressure (SBP) under 180 and diastolic blood pressure (DBP) under 105 mmHg throughout the MT procedure, without addressing the potential negative impacts of BP variability [1]. Furthermore, the 2014 guidelines from the Society of Neurointerventional Surgery and the Neurocritical Care Society recommend maintaining SBP above 140 mmHg [10]. Despite various studies suggesting the significance of personalized BP monitoring, none has yet demonstrated the efficacy of this approach [5, 8, 11]. 

Starting from February 2022, a BP management protocol was introduced at our university hospital center to enhance hemodynamic care for patients undergoing MT under GA.

The objective of this study was to evaluate the effectiveness of this individualized BP management protocol during MT under GA. We aimed to analyze its impact on procedural arterial hypotension and functional outcomes at 90 days. This was achieved by comparing the patient populations before and after the protocol’s implementation.

## Materials and methods

### Study design and subjects

We conducted a before-after study involving two retrospective cohorts of patients who underwent MT for AIS under GA at Angers University Hospital. The “before” group was composed of all patients included from January 2018 to June 2021 without the individualized BP management protocol. The “after” group included all patients treated from February 2022, following the implementation of the BP protocol, until February 2023.

The inclusion criteria encompassed adult patients (≥ 18 years old) with AIS caused by LVO diagnosed through cerebral imaging (all patients underwent a non-contrast CT scan and a CT angiography (CTA) of the supra-aortic vessels at admission. In cases where MRI was performed, standard sequences were acquired without perfusion imaging), and undergoing MT under GA. Exclusion criteria consisted of patients with unknown initial BP measurement, lacking automatic or computerized anesthetic monitoring, patients who had complete recanalization from IV thrombolysis prior to MT and thus did not undergo thrombectomy, individuals who experienced AIS subsequent to a cardiovascular procedure or recent GA (within 72 h), patients intubated before revascularization, those with a pre-procedural modified Rankin Scale (mRS) score above 2 [12] or unavailable, if revascularization was unsuccessful (TICI 0 and 1), or if there was no available data on neurological outcomes at 3 months.

### Recorded data

This study is a retrospective observational analysis based on prospectively collected data from the ETIS national registry, which includes all core clinical and procedural variables. Anesthetic management details and periprocedural blood pressure measurements, not available in the registry, were retrospectively extracted from the center’s electronic medical records. All data were systematically recorded and manually verified for accuracy.

We used data from the Endovascular Treatment in Ischemic Stroke Registry (ETIS) and from patient records. Baseline clinical data encompassed various aspects, including demographics (age, sex), comorbid conditions, and risk factors (smoking status, diabetes mellitus, chronic arterial hypertension, dyslipidemia, atrial fibrillation, prior ischemic coronary arterial disease, and previous ischemic stroke). Additionally, information was collected on anti-hypertensive and anticoagulant treatments, baseline mRS score, baseline NIHSS [13], pretreatment with IVT.

The characteristics of the stroke were described, comprising the occlusion site (such as terminal internal carotid, middle cerebral artery localization, tandem, isolated cervical carotid artery, basilar artery), the severity assessed using the Alberta Stroke Program Early CT Score (ASPECTS) based on non-enhanced CT brain imaging (NECT) or magnetic resonance imaging (MRI) [14], as well as the time intervals from symptom onset to groin puncture and revascularization.

Parameters related to the MT procedure were gathered, including the Revised Thrombolysis in Cerebral Infarction (mTICI) score assessed at the conclusion of the procedure [15], the count of passes made, the successful MT strategy employed (stent retriever, direct aspiration, combined technique), and any associated complications.

Biological data obtained upon admission were gathered, including parameters like blood glucose levels, hemoglobin concentrations, platelet counts, prothrombin time, and international normalized ratio.

All patients underwent a 24-hour NECT to evaluate potential complications such as intracranial hemorrhage (ICH). We also examined the incidence of symptomatic intracranial hemorrhage (SICH), defined as hemorrhage apparent on CT or MRI that correlated with a rise in the NIHSS score of ≥ 4 (as per ECASS II) within 7 days. Additionally, the ASPECT and NIHSS scores at 24 h were recorded.

We outlined the specific anesthetic agents employed for induction and the ones utilized for maintaining GA. Blood pressure data, including SBP, DBP and MAP, were measured prior to the induction of anesthesia, serving as the baseline BP. BP measurements were taken at intervals of every 5 min until 90 min following the commencement of the procedure. These values were automatically captured by the computerized anesthesia monitoring system. We documented the administration of vasoactive therapies like nicardipine, urapidil, noradrenaline (NAD), neosynephrine, or ephedrine, along with their respective dosages.

### Ethical considerations

The study protocol and consent form received approval from the Ethics Committee of Angers University Hospital (2023-026). In accordance with French Bioethics regulations, oral informed consent was obtained from patients if their level of consciousness permitted, or from their relatives otherwise. Data confidentiality was strictly maintained in accordance with the guidelines of the French commission for data protection (Commission Nationale Informatique et Liberté, CNIL), with registration number ar23-0021v0. From the ETIS registry, the NCT number is NCT03776877.

### Blood pressure management

In the “before” group, BP management complied with current guidelines: maintaining BP below 180/105 mmHg (for SBP and DBP, respectively) during the MT procedure, following the 2019 AHA/ASA stroke guidelines [1] and maintaining SBP > 140 mmHg as outlined in the 2014 guidelines from the Society of Neurointerventional Surgery and the Neurocritical Care Society [10]. If an episode of hypotension occurred during the procedure, it was subsequently managed by administering a vasopressor agent (with the specific molecule and dosage determined by the anesthetist’s discretion). Any episode of what the treating anesthesiologist considered significant hypotension (for example, a > 20% drop in MAP or SBP < 140 if following the guideline threshold) was treated with vasopressors, but there was no uniform protocol or exact numeric trigger in that period.

In the “after” group, BP was managed in accordance with an individualized protocol (Supplemental Fig. 1A). Developed through collaboration between neuroradiologist and anesthetist experts from Angers University Hospital, this protocol was inspired by the DETERMINE study protocol [16]. Before MT, a BP cuff was positioned on the patient’s arm, tailored to their morphology, on the opposite side of the diluted NAD infusion. Baseline BP was obtained in the interventional radiology room prior to anesthesia induction. Throughout the procedure, BP was continuously monitored non-invasively every 5 min. To mitigate peri-procedural hypotension, diluted NAD (2 mg in 50 mL 0.9% NaCl solution, i.e. 40 µg/mL) was administered as a vasopressive support. NAD infusion commenced before induction at 0.2 mg/h using an electric syringe pump, and adjustments were made based on MAP changes.

During MT, the objective was to maintain MAP within 10% of baseline (i.e., −10% < MAP < 10%), with a minimal target MAP ≥ 80 mmHg. SBP and DBP were kept below 180/105 mmHg, and MAP was maintained below 130 mmHg.

In situations where MAP fell below the minimum target, NAD dosage was escalated incrementally by 0.04 to 0.08 mg/h. Conversely, if MAP exceeded the maximum target, NAD was reduced in increments of 0.04 to 0.08 mg/h. If initial arterial hypertension occurred (i.e., > 180/105 mmHg) or if hypertension persisted beyond the maximum target despite NAD discontinuation, intravenous nicardipine (0.5 mg/ml) was initiated using an electric syringe. It began at 1 mg/h for 5 min and was titrated by 0.5 mg/h every 5 min, without surpassing 5 mg/h. If this proved insufficient, urapidil (5 mg/ml) could be considered. Post-procedure, for cases of successful revascularization, the following BP targets were established: MAP ≥ 65 mmHg, SBP ≥ 120 mmHg and < 180 mmHg, DBP ≥ 40 mmHg and < 105 mmHg. In instances of unsuccessful revascularization, BP objectives would be determined through individual consultation with the neurologist.

### Outcomes

Neurological functional status at 90 days, determined using the mRS, was evaluated by either certified vascular neurologists during routine clinical visits or by a qualified study nurse via a standardized telephone interview. A favorable outcome was characterized by an mRS score ≤ 2, whereas a poor outcome was indicated by an mRS score > 2. This method is well described in the literature [17].

### Statistical analyses

Descriptive statistics were used to summarize baseline characteristics, procedural variables, and outcomes. Categorical variables were compared between the “before” and “after” groups using Chi-square or Fisher’s exact tests, as appropriate. Continuous variables were compared using Student’s t-test or the Mann–Whitney U test in case of non-normal distribution.

To compare the “before” and “after” phases regarding the primary endpoint (90-day mRS ≤ 2 vs. > 2), we used logistic regression models adjusted for empirically selected confounders (age, baseline NIHSS score, baseline ASPECTS, pre-induction MAP, revascularization duration, anticoagulant treatment, IVT, and occlusion site). Odds ratios (ORs) and their 95% confidence intervals (95% CI) were estimated.

We also described the intraprocedural BP course in both groups, including the proportion of patients experiencing MAP drops from baseline at different thresholds (5%, 10%, 15%, 20%, and 30%). These variables were analyzed descriptively and compared between groups.

All statistical analyses were conducted using R software (R Core Team, 2022, version 4.1.3). A p-value < 0.05 was considered statistically significant.

## Results

### Patients’ characteristics

#### Before and after groups

**Fig. 1:**
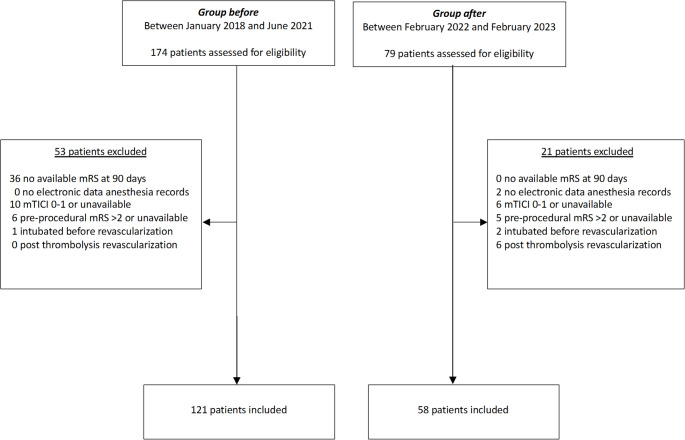
Flowchart of the study

A total of 179 patients were included in the final analysis, with 121 in the “before” group and 58 in the “after” group following protocol implementation. A total of 74 patients were excluded (53 from the “before” group and 21 from the “after” group), as depicted in Fig. 1.

The mean age of the cohort was 69.9 ± 14.8 years, with 89 patients (49.7%) being female. No significant differences emerged between the two groups in terms of baseline demographic and clinical characteristics. There were no noteworthy distinctions in baseline therapies, except for the use of CEI/A2R blockers: patients in the “after” group were more frequently treated with these blockers (45.6%) compared to the “before” group (28.1%) (*P* = 0.033). At baseline, no significant between-group differences were observed for mean NIHSS (16 ± 6 in the “before” group and 14 ± 7 in the “after” group) or mean ASPECTS (7 ± 2 and 6 ± 2 for the respective groups). A summary of patient characteristics is presented in Table 1. Comparison between included and excluded patients is summarized in (Supplemental Table 2).

**Table 1:** Population characteristics

		Overall population *n* = 179	Before group *n* = 121 (68)	After group *n* = 58 (32)	*P*
Age (years)		69.9 ± 14.8	69.3 ± 14.8	71.1 ± 14.9	0.427
Women		89 (49.7)	59 (48.8)	30 (51.7)	0.833
Wake up stroke		60 (33.5)	46 (38.0)	14 (24.1)	0.095
Hypertension		96 (53.6)	59 (48.8)	37 (63.8)	0.084
Dyslipidemia		66 (36.9)	45 (37.2)	21 (36.2)	1.000
Diabetes mellitus		21 (11.8)	12 (10.0)	9 (15.5)	0.287
Active smoking status		29 (16.9)	19 (16.5)	10 (17.5)	0.185
Acute ischemic stroke		14 (7.8)	10 (8.3)	4 (6.9)	0.983
Coronary heart disease		26 (14.5)	18 (14.9)	8 (13.8)	1.000
Atrial Fibrillation		28 (15.6)	18 (14.9)	10 (17.2)	0.851
CEI/A2R blockers		60 (33.7)	34 (28.1)	26 (45.6)	**0.033**
Beta blockers		63 (35.4)	41 (33.9)	22 (38.6)	0.656
Calcium channel blockers		22 (12.4)	17 (14.0)	5 (8.8)	0.451
Aspirin		37 (20.7)	25 (20.7)	12 (20.7)	1.000
Clopidogrel		9 (5.0)	5 (4.1)	4 (6.9)	0.670
Anticoagulation		23 (13)	16 (13.3)	7 (12.3)	0.715
Pre-TM mRS					0.304
	0	155 (86.6)	108 (89.3)	47 (81.0)	
	1	19 (10.6)	10 (8.3)	9 (15.5)	
	2	5 (2.8)	3 (2.5)	2 (3.4)	
Baseline NIHSS		16 ± 7	16 ± 6	14 ± 7	0.057
NIHSS after 24 h		12 ± 8	12 ± 9	13 ± 7	0.608
Thrombolysis		87 (48.6)	64 (52.9)	23 (39.7)	0.134
Initial imagery					1.000
	MRI	153 (85.5)	103 (85.1)	50 (86.2)	
	CT	26 (14.5)	18 (14.9)	8 (13.8)	
Occlusion localization					0.294
	Carotid terminus	13 (7.3)	6 (5.0)	7 (12.1)	
	MCA (M1 segment)	102 (57.0)	73 (60.3)	29 (50.0)	
	MCA (M2 segment)	33 (18.4)	24 (19.8)	9 (15.5)	
	Tandem	21 (11.7)	13 (10.7)	8 (13.8)	
	Cervical carotid	3 (1.7)	2 (1.7)	1 (1.7)	
	Basilar trunk	7 (3.9)	3 (2.5)	4 (6.9)	
MT procedure					**< 0.001**
	Stent retriever	24 (13.4)	24 (19.8)	0 (0.0)	
	Thromboaspiration	16 (8.9)	3 (2.5)	13 (22.4)	
	Combined technique	139 (77.7)	94 (77.7)	45 (77.6)	
Final TICI					0.214
	2a	13 (7.3)	8 (6.6)	5 (8.6)	
	2b	32 (17.9)	21 (17.4)	11 (18.9)	
	2c	53 (29.6)	31 (25.6)	22 (37.9)	
	3	81 (45.3)	61 (50.4)	20 (34.5)	
Glycemia (g/L)		1.35 ± 0.47	1.33 ± 0.51	1.39 ± 0.40	0.532
Hemoglobin (g/dL)		13.1 ± 2.3	13.1 ± 2.6	13.2 ± 1.8	0.905
Blood platelets (G/L)		234 ± 95	226 ± 93	245 ± 98	0.275
Thrombin time (%)		92 ± 21	92 ± 22	94 ± 20	0.610
Baseline ASPECTS		7 ± 2	7 ± 2	6 ± 2	0.121
ASPECTS after 24 h		6 ± 2	6 ± 2	6 ± 2	0.513
Onset to imagery time (min)		126 [95–163]	134 [105–171]	106 [86–147]	0.005
Onset to anesthesia time (min)		275 [210–330]	275 [214–331]	275 [208–347]	0.907
Onset to revascularization time (min)		322 [259–384]	318 [266–381]	337 [233–387]	0.524
Duration of procedure (min)		86 [60–120]	79 [59–114]	89 [71–122]	0.257
Duration of revascularization (min)		38 [25–60]	37 [24–61]	45 [27–59]	0.133
Periprocedural complications		13 (7.3)	8 (6.6)	5 (8.6)	0.859
Craniectomy		7 (4.0)	7 (5.9)	0 (0.0)	0.141
Hemorrhagic transformation		52 (29.4)	36 (30.0)	16 (28.1)	0.931
Symptomatic hemorrhagic transformation		12 (6.7)	8 (6.6)	4 (6.9)	0.943
mRS					0.242
	> 2	89 (49.7)	56 (46.3)	33 (56.9)	
	$\:\le\:\:$2	90 (50.3)	65 (53.7)	25 (43.1)	

*Count (%); mean ± standard deviation; median [interquartile range]*

*A2R Angiotensin II Receptor, ASPECTS Alberta Stroke Program Early CT Score, CEI Conversing Enzyme Inhibitor, CT Computed Tomography, MAP Mean Arterial Pressure, mRS modified Rankin Scale, MT Mechanical Thrombectomy, TICI Treatment in Cerebral Ischemia, MCA Middle Cerebral Artery, MRI Magnetic Resonance Imaging, NIHSS National Institutes of Health Stroke Scale*

The two groups did not show any significant differences in occlusion location, despite a higher rate of basilar (2.5% versus 6.9%), tandem occlusion (10.7% versus 13.8%) and carotid termination (5% versus 12.1%) in the “after” group treatment (*P* = 0.294). Mean time from symptom onset to groin puncture, mean time from symptom onset to recanalization, or mean procedure duration did not show any significant difference. In terms of the MT technique, there was a higher thromboaspiration rate in the “after” group (22.4%) compared to the “before” group (2.5%) (*P* < 0.001). Among the entire cohort, 166 patients (92.8%) achieved successful reperfusion (TICI score ≥ 2b), with 113 patients (93.4%) in the “before” group and 53 patients (91.3%) in the “after” group. No notable disparities were found in revascularization outcomes (*P* = 0.214).

#### Outcomes

Among the 179 patients, 90 individuals (50.3%) achieved a favorable outcome at 90 days (mRS ≤ 2): 65 patients (53.7%) in the “before” group and 25 patients (43.1%) in the “after” group (*P* = 0.242). Out of the total, 52 patients (29.4%) experienced ICH with 36 patients (30.0%) in the “before” group and 16 patients (28.1%) in the “after” group (*P* = 0.931). SICH occurred in 8 patients (6.6%) and 4 patients (6.9%), respectively (*P* = 0.943).

### BP and outcomes

#### BP in the two groups

**Fig. 2:**
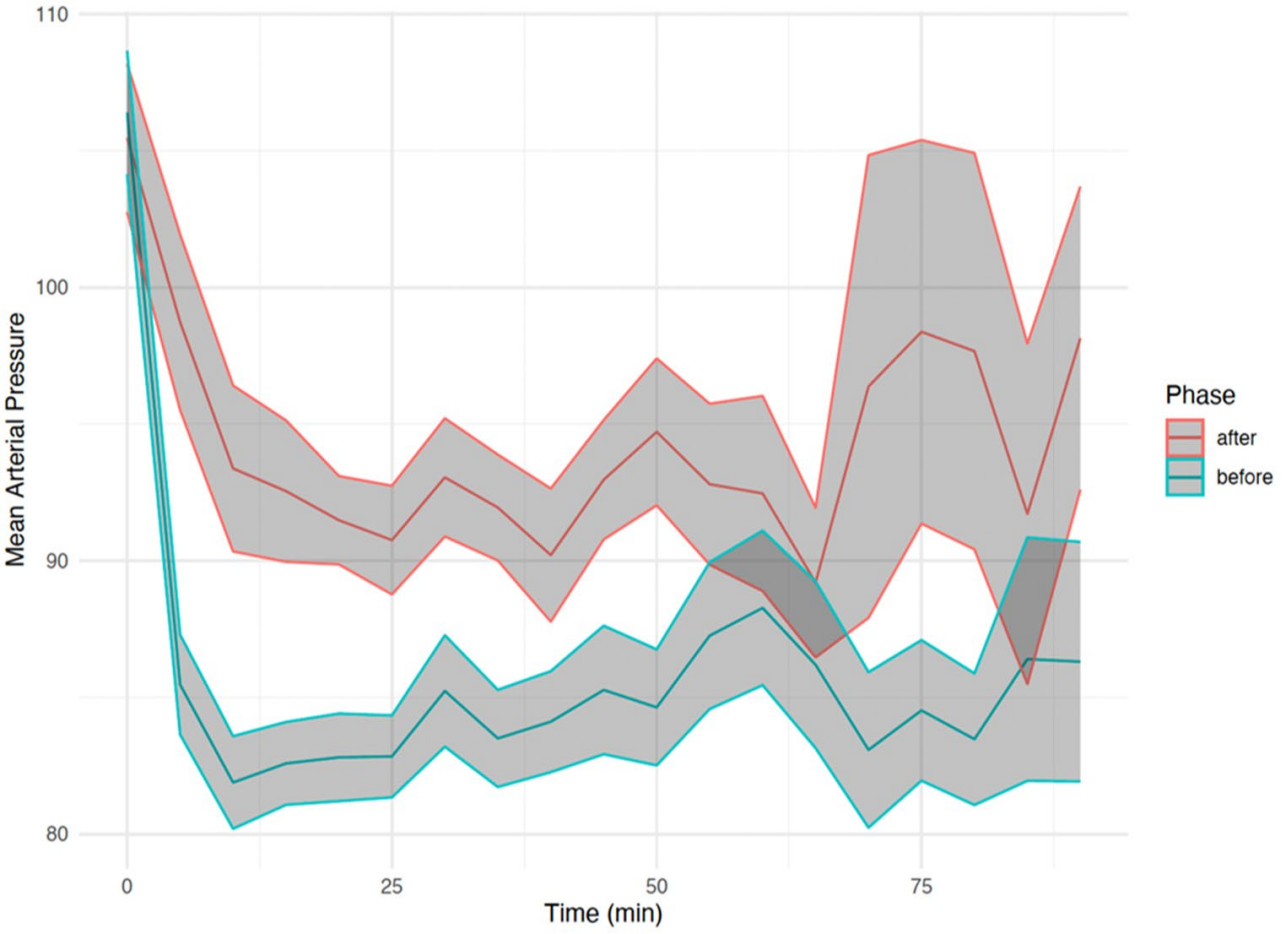
Evolution of procedural mean arterial blood pressure over time in the two groups Legend: This graph shows the average procedural MAP (y-axis) at regular time intervals (x-axis, in minutes) from induction of general anesthesia up to 90 min. The red line represents patients treated after the implementation of the blood pressure management protocol, while the blue line represents those treated before. Shaded areas around each line represent ± 1 standard error of the mean (SEM), reflecting interindividual variability. After an initial MAP drop in both groups immediately after induction, the “after” group maintained a higher and more stable MAP throughout the procedure compared to the “before” group. These data illustrate the effect of the individualized protocol in reducing the depth and duration of intra-procedural hypotension

No statistically significant differences were noted between the “before” and “after” groups in terms of baseline SBP (154 ± 30 vs. 150 ± 28, *P* = 0.397), baseline DBP (89 ± 27 vs. 87 ± 21, *P* = 0.709), and baseline MAP (106 ± 25 vs. 105 ± 21, *P* = 0.805). During the procedure, BP tended to be higher in the “after” group, with median MAP values exceeding 90 mmHg. The evolution of MAP over time in the two groups is shown in Fig. 2.

NAD usage was significantly more prevalent in the “after” group, with 65.8% in the “before” group and 94.8% in the “after” group (*P* < 0.001). The “before” group had a higher utilization of ephedrine at 38.8% compared to 6.9% in the “after” group (*P* < 0.001). Similarly, the use of neosynephrine was higher in the “before” group at 11.6% versus 0.0% in the “after” group (*P* = 0.016). Details about intra-procedural anesthetic management are summarized in Table 2.

**Table 2:** Intraprocedural anesthetic data

		*Overall population n* = 179	*Group before n* = 121	*Group after n* = 58	*P*-value
Maintenance anesthetic					0.051
	Propofol	148 (84.1)	96 (80.0)	52 (92.9)	
	Sevoflurane	28 (15.9)	24 (20.0)	4 (7.1)	
Pre-induction anti-hypertensive		5 (2.8)	4 (3.3)	1 (1.7)	0.907
Noradrenaline					
	Before induction	30 (16.9)	19 (15.8)	11 (19.0)	0.757
	During induction	100 (56.2)	53 (44.2)	47 (81.0)	**< 0.001**
	During procedure	134 (75.3)	79 (65.8)	55 (94.8)	**< 0.001**
Ephedrine during procedure		51 (28.5)	47 (38.8)	4 (6.9)	**< 0.001**
Neosynephrine during procedure		14 (7.8)	14 (11.6)	0 (0.0)	**0.016**
Pre-induction SBP (mmHg)		153 ± 29	154 ± 30	150 ± 28	0.397
Pre-induction DBP (mmHg)		88 ± 25	89 ± 27	87 ± 21	0.709
Pre-induction MAP (mmHg)		106 ± 24	106 ± 25	105 ± 21	0.805
MAP drops					
	5% from the baseline	142 (79.3)	97 (80.2)	45 (77.6)	0.840
	10% from the baseline	133 (74.3)	90 (74.4)	43 (74.1)	1.000
	15% from the baseline	124 (69.3)	85 (70.2)	39 (67.2)	0.814
	20% from the baseline	110 (61.5)	80 (66.1)	30 (51.7)	0.092
	30% from the baseline	78 (43.6)	59 (48.8)	19 (32.8)	0.063
Hypertension during procedure*		26 (14.5)	14 (11.6)	12 (20.7)	0.163

Count (%); mean ± standard deviation

*MAP* Mean Arterial Pressure, *SBP* Systolic Blood Pressure, *DBP* Diastolic Blood Pressure

* at least one episode of SBP > 180 mmHg

In the “before” group, 14 patients (11.6%) experienced at least one episode of hypertension (SBP > 180 mmHg) compared to 12 patients (20.7%) in the “after” group (*P* = 0.163). When examining both cohorts together, 142 patients (79.3%) encountered at least one MAP drop under 5%, 133 patients (74.3%) under 10% from baseline, 124 patients (69.3%) under 15% from baseline, 110 patients (61.5%) under 20% from baseline, and 78 patients (43.6%) under 30% from baseline.

Upon comparison between the “before” and “after” groups, MAP drops were less frequent in the “after” group, though the differences were not statistically significant: 80.2% versus 77.6% under 5% from baseline MAP (*P* = 0.840), 74.4% versus 74.1% under 10% from baseline (*P* = 1), 70.2% versus 67.2% under 15% from baseline (*P* = 0.814), 66.1% versus 51.7% under 20% from baseline (*P* = 0.092), and 48.8% versus 32.8% under 30% from baseline (*P* = 0.063).

### Comparing neurological outcome before and after the BP management protocol

In the univariate analysis, patients in the “after” group exhibited poorer neurological outcomes: 56 patients (46.3%) had a poor outcome in the “before” group, compared to 33 patients (56.9%) in the “after” group (*P* = 0.242). Table 1.

The results remained non-significant when controlling for age, NIHSS score, ASPECTS, pre-induction MAP, and revascularization duration (OR 1.59; 95% CI 0.72–3.60; *P* = 0.121). The association remained statistically non-significant after both reducing the list of confounding factors (namely, age, NIHSS score, and ASPECTS) and expanding it (including age, NIHSS score, ASPECTS, pre-induction MAP, revascularization duration, anticoagulant treatment, IVT, and occlusion localization). The results comparing neurological outcomes before and after the BP management protocol are outlined in Table 3. In three multivariate logistic regression models adjusting for increasing confounders—including age, NIHSS, ASPECTS, pre-induction MAP, procedure duration, anticoagulation, IVT, and occlusion site—the odds ratios for poor outcome (mRS > 2) in the ‘after’ (protocol) group compared to the ‘before’ group were 1.86 (95% CI 0.87–4.11, *p* = 0.115), 1.59 (0.72–3.60, *p* = 0.121), and 1.13 (0.45–2.83, *p* = 0.786), respectively; none reached statistical significance.

**Table 3:** Adjusted odds of poor outcome (mRS >2) for ‘after’ vs ‘before’ group under various models

	OR	95% CI	*P*-value
Model 1	1.86	0.87–4.11	0.115
Model 2	1.59	0.72–3.60	0.121
Model 3	1.13	0.45–2.83	0.786

*Model 1: adjusted for age*,* NIHSS score and ASPECTS*

*Model 2: adjusted for age*,* NIHSS score*,* ASPECTS*,* pre-induction MAP*,* and revascularization duration.*

*Model 3: adjusted for age*,* NIHSS score*,* ASPECTS*,* pre-induction MAP*,* revascularization duration*,* anticoagulant treatment*,* IVT*,* and occlusion localization.*

## Discussion

In this study, our objective was to assess the influence of an individualized BP management protocol on arterial hypotension and its impact on functional neurological outcomes at 3 months for patients undergoing MT under GA for AIS. After implementing the protocol, we observed a decrease in the occurrence of arterial hypotension and an increase in MAP during the procedure, indicating protocol effectiveness despite the absence of statistical significance. However, we did not find a significant association with favorable neurological outcomes at 3 months for the group subjected to the individualized protocol. There was even a trend towards worse neurological outcomes at 90 days, although this did not reach statistical significance in the multivariate analyses.

Other trials evaluated the effectiveness of personalized BP management during MT. The INDIVIDUATE study, a single-center randomized controlled trial, focused on maintaining intraprocedural SBP at the presented level, with a 10 mmHg margin. The 3-month outcomes showed no significant difference in favorable functional outcomes between individualized and standard BP management (25% vs. 24%; adjusted odds ratio, 0.81 [95% CI, 0.41–1.61]; *P* = 0.56) [18].

The DETERMINE trial, a randomized, controlled, multicenter study, aims to assess the clinical significance of individualized BP management during MT compared to international recommendations under GA or conscious sedation. Similar to our protocol, DETERMINE maintains MAP within 10% of the pre-procedural measurement, administering NAD systematically to prevent hypotension [16]. Their results have not been published yet.

Indeed, there are multiple interconnected hypotheses that might account for our findings. One potential explanation for these results is that efforts to minimize hypotensive incidents could have inadvertently led to elevated BP levels during the procedure, potentially producing an unintended counterproductive effect. This hypothesis was not confirmed in our study.

The observations from the “after” group indicate that MAP values during the procedure consistently remained elevated, surpassing 90 mmHg. A previous retrospective cohort study conducted by Rasmussen et al., which included 365 AIS patients, demonstrated an association between MAP levels exceeding 90 mmHg for over 45 min during MT and less favorable neurological outcomes at 3 months [19]. The consistently higher BP levels during the procedure could potentially have adverse effects, suggesting the possibility of a dose-response relationship for BP that follows a U-shaped curve.

Our findings are discussed in light of the recent multicenter study by Chang et al. (2025), which demonstrated improved outcomes with systolic blood pressure maintained above 130–140 mmHg during EVT. Although we did not observe a similar benefit, possibly due to sample size or design differences, these results support the clinical relevance of avoiding hypotension—one of the key targets of our protocol [20].

It is noteworthy that patients in the “after” group had a lower utilization of IVT, with only 39.7% receiving IVT compared to 52.9% in the “before” group. The presence of contraindications to IVT often identifies a subset of patients with higher rates of comorbidities, potentially including concurrent medical conditions, medications, or recent surgical history. This difference could contribute to the observed poorer neurological outcomes at 3 months. Existing research highlights the synergistic benefit of combining IVT and MT compared to MT alone. A recent meta-analysis of 30 clinical studies indicated that the bridging approach (IVT + MT) yields improved functional outcomes and reduced 90-day mortality without increasing the risk of early hemorrhagic complications [21]. This might be attributed to IVT’s potential for accelerating early reperfusion in the ischemic region and resolving residual distal thrombi. Such findings align with current recommendations [1] advocating for IVT before TM when it is clinically indicated.

Regarding the complications associated with MT, we did not specifically account for emboli occurring in the same area or in new territories. However, research has demonstrated that such embolic events resulting in new ischemic lesions are predictive factors for unfavorable functional outcomes and are among the most common complications following MT [22]. Recent studies utilizing diffusion-weighted imaging after MT have revealed that embolic events can manifest in approximately one-third of patients [23]. As a result, only a minority of these embolic complications can be detected angiographically, and their clinical significance appears to be linked to the size of the resulting ischemic lesion.

In relation to the techniques used in MT, there was a notably higher utilization of contact aspiration alone in the “after” group. However, it is worth mentioning the ASTER trial (The Contact Aspiration vs. Stent Retriever for Successful Revascularization), a multicenter randomized clinical trial that directly compared contact aspiration and the standard stent retriever technique for TM in AIS patients. This trial discovered no significant difference between the two techniques in terms of revascularization efficacy or neurological outcomes at 3 months [24]. Similarly, the multicenter randomized COMPASS trial also demonstrated that direct aspiration is non-inferior to stent retriever first-line thrombectomy in terms of neurological outcomes at 90 days [25]. These findings provide substantial support for considering direct aspiration as a viable alternative to stent retrievers for MT in AIS. In contrast to earlier reports, the VECTOR trial—a randomized, single‑blind, multicenter study of 521 patients—found no significant difference in successful reperfusion (eTICI 2c–3) or clinical outcomes between a combined technique (stent retriever plus contact aspiration) and contact aspiration alone in anterior circulation LVO with susceptibility vessel sign (58% vs. 52%; OR 1.27, 95% CI 0.88–1.83; *p* = 0.19) [26].

A before-after study has certain limitations. Throughout the analyzed period, various factors before, during, or after the procedure may have changed. Despite evaluating many factors, attributing results solely to the protocol is challenging. Our single-centre study, which included a limited number of patients, may have reduced both the statistical power and the generalisability of our findings. The retrospective data collection could lead to data loss and unaccounted confounding variables. The BP data collection was carried out automatically and via computerized means; however, the data points were recorded at intervals of every 5 min, which might diminish the accuracy in capturing rapid BP variations. Moreover, BP measurements were acquired non-invasively through the use of cuffs, which tends to be less precise compared to invasive BP measurements. Nevertheless, the literature recommends not wasting time setting up invasive blood pressure measurement in the treatment of stroke by thrombectomy [27]. Although not statistically significant, the differences in occlusion sites may indicate potentially more severe strokes in the “after” group. However, this variable was included in the multivariate model and did not affect the results.

Given the study’s design and the limited patient population, extrapolating the findings to a broader context can be difficult. Therefore, it is advisable to await the outcomes of randomized trials investigating the effects of individualized BP monitoring during MT. These trials would provide more robust evidence and insights into the potential benefits of such an approach.

## Conclusion

Our study indicated that the implementation of individualized BP management resulted in decreased drops in MAP compared to baseline, particularly for cases of severe hypotension. However, our findings did not reveal any favorable impact of the BP management protocol on neurological outcomes at 3 months.

## Supplementary Information

Below is the link to the electronic supplementary material.

Supplementary material 1.

## Data Availability

All anonymised data and statistics are available for checking.

## Abbreviations

AIS: Acute Ischemic Stroke
ASPECTS: Alberta Stroke Program Early CT Score
BP: Blood Pressure
DBP: Diastolic Blood Pressure
GA: General Anesthesia
ICH: Intracranial Hemorrhage
IVT: Intravenous Thrombolysis
LVO: Large Vessel Occlusion
MAP: Mean Arterial Pressure
MRI: Magnetic Resonance Imaging
mRS: Modified Rankin Scale
MT: Mechanical Thrombectomy
NAD: Noradrenaline
NECT: Non-contrast Enhanced Computed Tomography
NIHSS: National Institutes of Health Stroke Scale
mTICI: Modified Thrombolysis in Cerebral Infarction
SBP: Systolic Blood Pressure
