# Supplementary material for: Effect of personalized blood pressure management during mechanical thrombectomy under general anesthesia: a single-center before–after study
Source: Neuroradiology. 2026 Jan 14;68(5):1137–47. doi: 10.1007/s00234-025-03878-6 (PMC13216165; doi:10.1007/s00234-025-03878-6)
Supplement: Supplementary file 1 — Supplementary material 1. [file 234_2025_3878_MOESM1_ESM.docx]

**Supplemental materials**

**Figure 1A** Local procedure for blood pressure management during thrombectomy under general anesthesia at the university hospital of Angers.


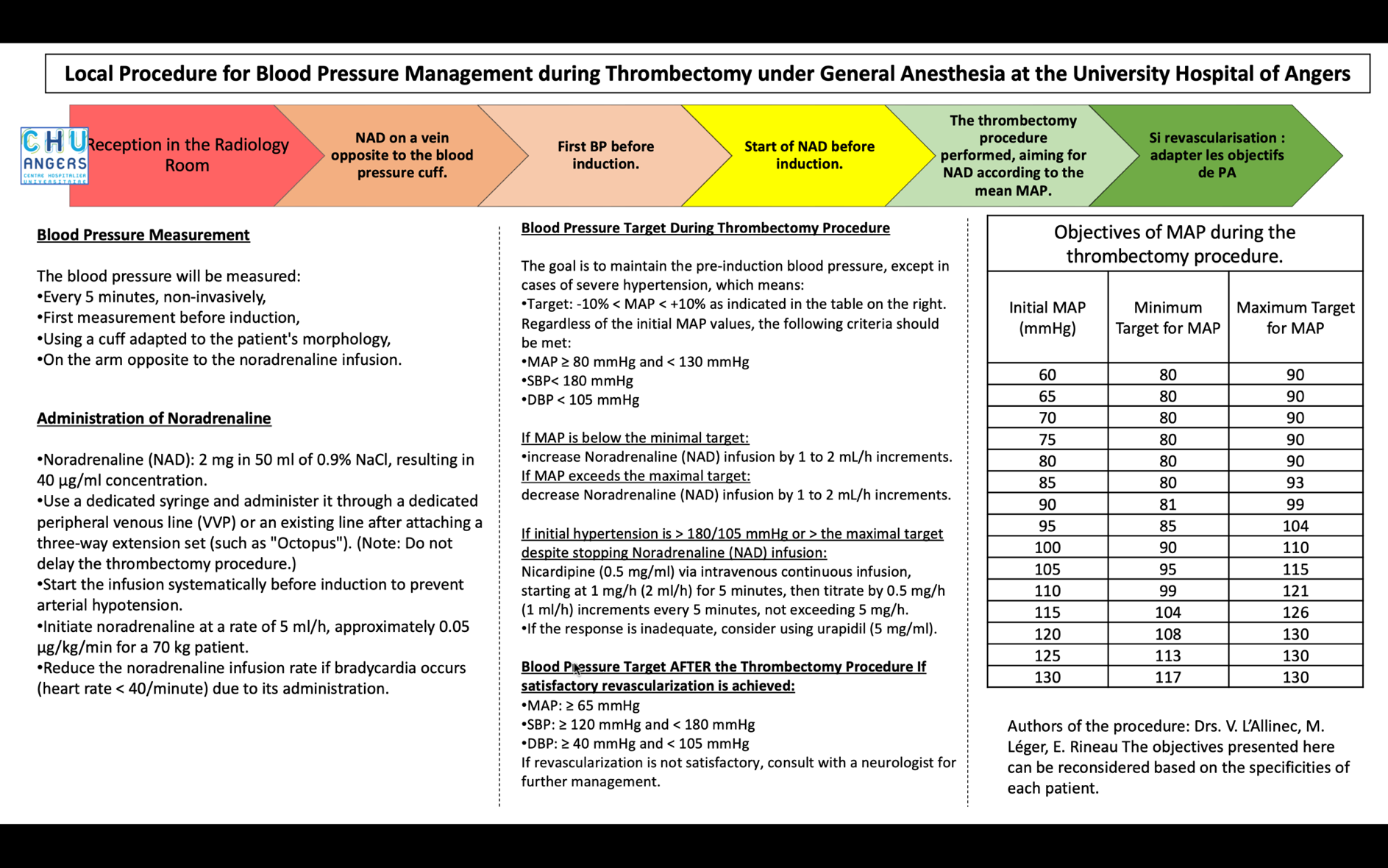


**Table 2A** Comparison between included and excluded patients in clinical, biological and radiological characteristics

|  |  | Study cohort  n = 179 | Excluded patients  n = 74 | P value |
| --- | --- | --- | --- | --- |
| Phase | before | 121 (67.6) | 53 (71.6) | 0.632 |
|  | after | 58 (32.4) | 21 (28.4) |  |
| *Clinical features* |  |  |  |  |
| Age |  | 69.9 ± 14.8 | 74.5 ± 13.1 | 0.021 |
| Women |  | 89 (49.7) | 39 (52.7) | 0.769 |
| Wake up stroke |  | 60 (33.5) | 21 (31.3) | 0.864 |
| Hypertension |  | 96 (53.6) | 51 (69.9) | 0.026 |
| Dyslipidemia |  | 66 (36.9) | 26 (35.6) | 0.965 |
| Diabete Mellitus |  | 21 (11.8) | 8 (11.0) | 0.522 |
| Smoking |  | 29 (16.9) | 3 (4.3) | 0.029 |
| Acute ischemic stroke |  | 14 (7.8) | 8 (11.0) | 0.579 |
| Coronary heart disease |  | 26 (14.5) | 9 (12.3) | 0.798 |
| Atrial fibrillation |  | 28 (15.6) | 18 (24.7) | 0.133 |
| CEI/A2R blockers |  | 60 (33.7) | 30 (46.2) | 0.103 |
| Beta-blockers |  | 63 (35.4) | 34 (52.3) | 0.025 |
| Calcium channel blockers |  | 22 (12.4) | 8 (12.3) | 1.000 |
| Aspirin |  | 37 (20.7) | 8 (12.5) | 0.209 |
| Clopidogrel |  | 9 (5.0) | 4 (6.2) | 0.961 |
| Anticoagulation |  | 25 (13) | 26 (27.3) | 0.077 |
| *Biological features* |  |  |  |  |
| Glycaemia (g/L) |  | 1.35 ± 0.47 | 1.29 ± 0.29 | 0.385 |
| Hemoglobin (g/dL) |  | 13.2 ± 2.3 | 12.7 ± 3.4 | 0.367 |
| Blood platelets (G/L) |  | 234 ± 95 | 228 ± 92 | 0.722 |
| Thrombin time (%) |  | 92 ± 21 | 85 ± 23 | 0.057 |
| Baseline MAP (mmHg) |  | 104 ± 18 | 97 ± 22 | 0.015 |
| Pre-MT mRS | 0 | 155 (86.6) | 56 (81.2) | <0.001 |
|  | 1 | 19 (10.6) | 3 (4.3) |  |
|  | 2 | 5 (2.8) | 1 (1.4) |  |
|  | 3 | 0 (0.0) | 8 (11.6) |  |
|  | 4 | 0 (0.0) | 1 (1.4) |  |
| Intravenous thrombolysis |  | 87 (48.6) | 33 (45.8) | 0.797 |
| Baseline NIHSS |  | 16 ± 7 | 14 ± 7 | 0.168 |
| NIHSS at 24 hours |  | 12 ± 8 | 13 ± 9 | 0.555 |
| *Radiological features* |  |  |  |  |
| Initial imagery | MRI | 153 (85.5) | 61 (82.4) | 0.676 |
|  | CT | 26 (14.5) | 13 (17.6) |  |
| Occlusion localization | Carotid terminus | 13 (7.3) | 5 (6.8) | 0.115 |
|  | MCA (M1 segment) | 102 (57.0) | 42 (56.8) |  |
|  | MCA (M2 segment) | 33 (18.4) | 10 (13.5) |  |
|  | Tandem | 21 (11.7) | 5 (6.8) |  |
|  | Cervical carotid | 3 (1.7) | 4 (5.4) |  |
|  | Basilar trunk | 7 (3.9) | 8 (10.8) |  |
| Baseline ASPECTS | 0 | 0 (0.0) | 1 (1.4) | 0.674 |
|  | 1 | 2 (1.1) | 1 (1.4) |  |
|  | 2 | 4 (2.3) | 3 (4.3) |  |
|  | 3 | 10 (5.6) | 2 (2.9) |  |
|  | 4 | 10 (5.6) | 4 (5.8) |  |
|  | 5 | 20 (11.3) | 5 (7.2) |  |
|  | 6 | 25 (14.1) | 7 (10.1) |  |
|  | 7 | 41 (23.2) | 14 (20.3) |  |
|  | 8 | 34 (19.2) | 15 (21.7) |  |
|  | 9 | 19 (10.7) | 9 (13.0) |  |
|  | 10 | 12 (6.8) | 8 (11.6) |  |
| ASPECTS after 24 hours | 0 | 5 (2.1) | 3 (1.7) | 0.042 |
|  | 1 | 8 (3.3) | 5 (2.8) |  |
|  | 2 | 11 (4.6) | 11 (6.2) |  |
|  | 3 | 23 (9.6) | 12 (6.8) |  |
|  | 4 | 21 (8.8) | 20 (11.3) |  |
|  | 5 | 22 (9.2) | 19 (10.7) |  |
|  | 6 | 34 (14.2) | 25 (14.1) |  |
|  | 7 | 45 (18.8) | 34 (19.2) |  |
|  | 8 | 40 (16.7) | 28 (15.8) |  |
|  | 9 | 20 (8.4) | 14 (7.9) |  |
|  | 10 | 10 (4.2) | 6 (3.4) |  |
| Final TICI | 0 | 0 (0.0) | 11 (17.2) | <0.001 |
|  | 1 | 0 (0.0) | 4 (6.2) |  |
|  | 2a | 13 (7.3) | 2 (3.1) |  |
|  | 2b | 32 (17.9) | 6 (9.3) |  |
|  | 2c | 53 (29.6) | 14 (21.8) |  |
|  | 3 | 81 (45.3) | 27 (42.2) |  |
| Perprocedural complications |  | 13 (7.3) | 7 (10.6) | 0.559 |
| Craniectomy |  | 7 (4.0) | 3 (4.5) | 1.000 |
| Hemorrhagic transformation |  | 52 (29.4) | 21 (30.9) | 0.941 |

*Count (%) ; mean ± standard deviation ; median [interquartile range]*

*A2R, Angiotensin II Receptor; ASPECTS, Alberta Stroke Program Early CT Score; CEI, Conversing Enzyme Inhibitor; CT, Computed Tomography; MAP, Mean Arterial Pressure; MT, Mechanical Thrombectomy; TICI, Treatment in Cerebral Ischemia; MCA, Middle Cerebral Artery; MRI, Magnetic Resonance Imaging; NIHSS, National Institutes of Health Stroke Scale*
